# Combining HLA-DRB1-DQB1 and *Mycobacterium Avium Subspecies Paratubercolosis* (MAP) antibodies in Sardinian multiple sclerosis patients: associated or independent risk factors?

**DOI:** 10.1186/s12883-016-0669-1

**Published:** 2016-08-23

**Authors:** J. Frau, D. Cossu, C. Sardu, G. Mameli, G. Coghe, L. Lorefice, G. Fenu, S. Tranquilli, L. A. Sechi, M. G. Marrosu, E. Cocco

**Affiliations:** 1 Multiple Sclerosis Centre, Department of Public Health, Clinical and Molecular Medicine, University of Cagliari, Cagliari, Italy; 2 Department of Biomedical Sciences, Section of Experimental and Clinical Microbiology, University of Sassari, Sassari, Italy; 3 Department of Public Health, Clinical and Molecular Medicine, University of Cagliari, Cagliari, Italy; 4 Multiple Sclerosis Centre, Department of medical sciences, University of Cagliari, Cagliari, Italy

**Keywords:** *Mycobacterium avium subspecies paratuberculosis*, Multiple sclerosis, HLA, Sardinia

## Abstract

**Background:**

Amongst Sardinians the human leukocyte antigen (HLA) DRB1-DQB1 haplotypes *15:02-*06:01, *16:01-*05:02, *14:01-4-*05:03 are protective for multiple sclerosis (MS), while *13:03-*03:01, *04:05-*03:01, *03:01-*02:01, *15:01-*06:02 and *Mycobacterium avium subspecies paratubercolosis (MAP)* are predisposing factors. We studied the correlation between MAP and HLA.

**Methods:**

Five hundred thirty-one patients were searched for anti-MAP2694 antibodies, DRB1-DQB1 genotyping was performed. The haplotypes were classified as predisposing, neutral or protective.

**Results:**

Anti-MAP2694 were found in 23 % of subjects carrying one protective HLA versus 32 % without (*p* = 0.04).

**Conclusions:**

We showed a lower frequency of Abs in patients with protective HLA. These haplotypes could have a protective role for both MS and MAP.

## Background

Multiple Sclerosis (MS) is a multifactorial disease shaped by both genetic and environmental factors. In Sardinia, an Italian island with a very high prevalence of the disease, the genetics of MS have some peculiarities. In particular, there is a negative association with the DRB1-DQB1 human leukocyte antigen (HLA) haplotypes *15:02-*06:01, *16:01-*05:02 and *14:01-4-*05:03 and a positive association with *13:03-*03:01, *04:05-*03:01, *03:01-*02:01 and *15:01-*06:02 [1]. The last of these is the main genetic risk factor for MS in Northern European populations, [2] it gives Sardinians a strong predisposition to MS, but is very rare in this population [3]. Concerning environmental factors, an association between MS and *Mycobacterium avium subspecies paratubercolosis* (MAP) was identified on the island [4]. MAP is an endocellular pathogen, it is present in 39.2 % of patients and in 6 % of healthy controls (HC) in the Sardinian population. It could be a trigger for the disease by means of a molecular mimicry mechanism and the spreading of epitopes [5].

In recent years, many studies have focused on the possibility of a relationship between genetic and infectious factors in conferring risk of MS, in particular between predisposing HLA and Epstein-Barr virus (EBV) [6–8]. The studies yielded conflicting results (negative interaction in the first study, and positive in two others). Other studies aimed to investigate the influence of genotype on levels of humoral response to EBV. Waubant et al. showed higher levels of EBNA-1 antibodies (Abs) in HLA-DRB1 positive patients [9]. Rubicz et al. used a genome-wide approach to analyze the SNPs associated with EBV-related diseases, finding an association between EBNA-1 Abs levels and HLA [8].

There are no studies focused on the relationship between HLA and MAP.

We aimed to search for possible relationships between the presence of anti-MAP Abs and predisposing/protective HLA in MS patients.

## Methods

A group of MS patients, diagnosed according to McDonald criteria 2010 [10], was enrolled in the study after giving informed written consent. They were recruited randomly from the MS center of the University of Cagliari, between patients performing routine visits. The only inclusion criterion was to have a diagnosis of MS (McDonald 2010) and there were not exclusion criteria. In fact, in a previous study, we did not find association between presence of anti-MAP antibodies and clinical variables [5]. The University of Cagliari ethical committee approved the study and the informed consent. The clinical and demographic variables are reported in Table 1. For each subject a blood sample was collected and analyzed for the presence of anti-MAP2694 Abs by indirect ELISA, as previously described [4]. Moreover, DNA was extracted to perform DRB1-DQB1 HLA genotyping and the haplotypes were classified as predisposing (*13:03-*03:01, *04:05-*03:01, *03:01-*02:01, *15:01-*06:02) or protective (*15:02-*06:01, *16:01-*05:02, *14:01-4-*05:03), according to the previous study [1]. Other haplotypes were considered neutral.

**Table 1:** Clinical and demographic data of patients included in the study

	Mean age	Median EDSS	Course			Therapy							Steroids
			RR	SP	PP	NONE	IFN	GA	NAT	FING	MIX	AZA	
ALL	43 y	2.5	82 %	13 %	5 %	31 %	25 %	18 %	10 %	12 %	1 %	3 %	6 %
MAP +	41 y	2	81 %	15 %	4 %	29 %	27 %	17 %	10 %	13 %	0 %	4 %	5 %
MAP-	44 y	2.5	84 %	10 %	6 %	32 %	26 %	16 %	11 %	13 %	1 %	1 %	6 %

*RR* relapsing remitting, *SP* secondary progressive, *PP* primary progressive, *IFN* beta interferon, *GA* glatiramer acetate, *NAT* natalizumab, *FING* fingolimod, *MIX* mitoxantrone, *AZA* azathioprine

### Statistical analysis

The Pearson’s chi squared test was used for the haplotypes and genotypes analysis. For the former, protective haplotypes versus neutral and predisposing were considered. The analysis was repeated considering neutral and predisposing haplotypes together. In the genotypes analysis, the presence of at least one protective haplotype versus its absence was evaluated.

## Results

The study enrolled 531 patients, 372 females and 159 males (F:M = 2.3:1). MAP negative subjects carried a total of 109 (15 %) protective, 317 (44 %) neutral and 296 (41 %) predisposing HLA haplotypes, while MAP positive patients carried a total of 33 (11 %) protective, 140 (46 %) neutral and 133 (43 %) predisposing HLA haplotypes (*p* = 0.19) Table 2. When considering protective HLA versus combined neutral and predisposing ones, MAP negative patients carried 613 (85 %) neutral or predisposing and 109 (15 %) protective HLA haplotypes, while MAP positive subjects carried 273 (89 %) neutral or predisposing and 33 (11 %) protective haplotypes (*p* = 0.07) Table 3.

**Table 2:** Analysis of haplotypes: protective versus neutral versus predisposing. Pearson’s chi squared (*p* = 0.19)

		Protective haplotypes	Neutral haplotypes	Predisposing haplotypes
Anti-MAP2694 Abs	Negative	109 (15 %)	317 (44 %)	296 (41 %)
	Positive	33 (11 %)	140 (46 %)	133 (43 %)

**Table 3:** Analysis of haplotypes: protective versus combined neutral and predisposing. Pearson’s chi squared (*p* = 0.07)

		Neutral or predisposing haplotypes	Protective haplotypes
Anti-MAP2694 Abs	Negative	613 (85 %)	109 (15 %)
	Positive	273 (89 %)	33 (11 %)

The analysis of the genotypes showed that the protective alleles were carried by 134 patients. The number of patients carrying at least one protective haplotype was 31 (20 %) in MAP positive and 103 (29 %) in MAP negative patients (*p* = 0.05) Table 4.

**Table 4:** Analysis of genotypes: Absence versus presence of at least one protective haplotype. Pearson’s chi squared (*p* = 0.05)

		Absence of protective haplotypes	At least one protective haplotypes
Anti-MAP2694 Abs	Negative	258 (71 %)	103 (29 %)
	Positive	122 (80 %)	31 (20 %)

## Discussion

Sardinian MS patients have some genetic and environmental peculiarities. They carried predisposing and protective haplotypes different from those carried by other MS populations [1]. Moreover, nowadays, it is only in Sardinia that the disease is associated with MAP infection [4, 5]. An interesting field of research is the possible interaction between the HLA haplotypes and the presence of anti-MAP Abs in conferring MS risk. Recently, a molecular modeling approach was used to find the differences between the Sardinian predisposing (*03:01 and *15:01) and protective (*16:01 and *15:02) HLA in recognizing MAP peptide. The former were found able to bind the MAP peptide better than the protective ones, suggesting a higher presentation efficiency, a higher probability of T-cell activation and autoimmune reaction [11].

In our study the analysis of the haplotypes showed no significant difference in frequency of predisposing, protective and neutral haplotypes among MAP+ and MAP- patients, suggesting that MAP infection and predisposing haplotypes are independent risk factors. This is in line with the study of De Jager et al., that analyzed the association between EBV infection, evaluating the levels of anti-EBNA-1 Abs and the presence of DRB1*1501 [6]. They concluded that both factors independently contributed to an increased MS risk. In their study no predisposing HLA were considered [6].

On the other hand, Rubicz et al. analyzed 30 genome-wide significant MS SNPs and found an association between EBNA-1 quantitative trait and SNP rs9271366, and between EBNA-1 serostatus trait (positive or negative) and SNPs rs3129860 and rs3135388. All these SNPs are located within HLA-DR and HLA-DQ genes, suggesting that this region is important both for EBV-related and autoimmune responses [8].

Sunqvist et al. found an interaction between EBNA: 385–420 IgG and the presence of DRB1*15. They concluded that the mechanism through which HLA influences MS risk could involve the immune control of EBV infection. They also evaluated the protective haplotype A*02, demonstrating the interaction between its absence and the presence of the same Abs [7].

This last point is in line with the findings in our study. In fact, the analysis of haplotypes identified a trend towards a lower frequency of Abs in patients with protective haplotypes. The analysis of genotypes confirmed this finding, showing a significantly lower presence of anti-MAP Abs in patients carrying at least one protective HLA versus patients without.

We did not find an association between the presence of Abs and predisposing HLA.

## Conclusions

In conclusion, on the basis of our data, we could hypothesize that both genetic and MAP have an independent role in conferring MS risk.

Moreover, considering the demonstration of lower frequency of anti-MAP Abs in patients with protective HLA, it is possible that these haplotypes play a role in protecting not only against MS but against MAP infection as well.

## Abbreviations

Abs: Antibodies
EBV: Epstein-barr virus
HC: Healthy controls
HLA: Human leukocyte antigen
MS: Multiple sclerosis
MAP: Mycobacterium avium subspecies paratubercolosis
